# Evaluation of the Privacy Risks of Personal Health Identifiers and Quasi-Identifiers in a Distributed Research Network: Development and Validation Study

**DOI:** 10.2196/24940

**Published:** 2021-05-31

**Authors:** SeHee Oh, MinDong Sung, Yumie Rhee, Namki Hong, Yu Rang Park

**Affiliations:** 1 Department of Biomedical Systems Informatics Yonsei University College of Medicine Seoul Republic of Korea; 2 Department of Internal Medicine Endocrine Research Institute Yonsei University College of Medicine Seoul Republic of Korea

**Keywords:** distributed research network, Observational Medical Outcomes Partnership common data model, privacy risk quantification, personal health identifier, quasi-identifier

## Abstract

**Background:**

Privacy should be protected in medical data that include patient information. A distributed research network (DRN) is one of the challenges in privacy protection and in the encouragement of multi-institutional clinical research. A DRN standardizes multi-institutional data into a common structure and terminology called a common data model (CDM), and it only shares analysis results. It is necessary to measure how a DRN protects patient information privacy even without sharing data in practice.

**Objective:**

This study aimed to quantify the privacy risk of a DRN by comparing different deidentification levels focusing on personal health identifiers (PHIs) and quasi-identifiers (QIs).

**Methods:**

We detected PHIs and QIs in an Observational Medical Outcomes Partnership (OMOP) CDM as threatening privacy, based on 18 Health Insurance Portability and Accountability Act of 1996 (HIPPA) identifiers and previous studies. To compare the privacy risk according to the different privacy policies, we generated limited and safe harbor data sets based on 16 PHIs and 12 QIs as threatening privacy from the Synthetic Public Use File 5 Percent (SynPUF5PCT) data set, which is a public data set of the OMOP CDM. With minimum cell size and equivalence class methods, we measured the privacy risk reduction with a trust differential gap obtained by comparing the two data sets. We also measured the gap in randomly sampled records from the two data sets to adjust the number of PHI or QI records.

**Results:**

The gaps averaged 31.448% and 73.798% for PHIs and QIs, respectively, with a minimum cell size of one, which represents a unique record in a data set. Among PHIs, the national provider identifier had the highest gap of 71.236% (71.244% and 0.007% in the limited and safe harbor data sets, respectively). The maximum size of the equivalence class, which has the largest size of an indistinguishable set of records, averaged 771. In 1000 random samples of PHIs, Device_exposure_start_date had the highest gap of 33.730% (87.705% and 53.975% in the data sets). Among QIs, Death had the highest gap of 99.212% (99.997% and 0.784% in the data sets). In 1000, 10,000, and 100,000 random samples of QIs, Device_treatment had the highest gaps of 12.980% (99.980% and 87.000% in the data sets), 60.118% (99.831% and 39.713%), and 93.597% (98.805% and 5.207%), respectively, and in 1 million random samples, Death had the highest gap of 99.063% (99.998% and 0.934% in the data sets).

**Conclusions:**

In this study, we verified and quantified the privacy risk of PHIs and QIs in the DRN. Although this study used limited PHIs and QIs for verification, the privacy limitations found in this study could be used as a quality measurement index for deidentification of multi-institutional collaboration research, thereby increasing DRN safety.

## Introduction

As medical data include sensitive personal patient information, various challenges are being studied to protect patient information and optimize research results, including artificial intelligence, federated learning, and distributed research networks (DRNs) [1-11]. Among the above challenges, the DRN is a multi-institutional collaboration network [1] for standardizing the data of participating institutions into a common structure, terminology, and software called a common data model (CDM) [12-16]. In such research networks, data are not shared directly, and only analysis results are shared [1,3,6,17]. In research where sharing sensitive patient information has limitations or where large-scale data privacy needs to be preserved, the DRN structure is applied to standardize the data, terminology, and software [4-6]. There are several CDMs in DRNs, including the Observational Medical Outcomes Partnership (OMOP) CDM of Observational Health Data Sciences and Informatics (OHDSI), Sentinel CDM of the Food and Drug Administration, and Patient‐Centered Outcomes Research Network of the Patient-Centered Outcomes Research Institute [18,19].

A DRN was recently recognized as a platform for protecting large-scale data [16,20-22]. DRN-based studies have argued two factors that enable the DRN infrastructure to mitigate privacy issues relative to other data sharing–based studies [1,6,23-29]. First, a DRN process protects patient information without directly sharing data [1,3,6,17]. Second, a CDM structure excludes some direct identifiers that could threaten the privacy of patient information, such as names and exact birthdays, by complying with the Health Insurance Portability and Accountability Act (HIPAA) [30-33]. Therefore, a DRN protects patient information through processes and structures.

However, previous studies have revealed limitations of DRNs in terms of data privacy. First, a DRN in a single site has privacy issues similar to a conventional database owing to repeated reuse [34-41]. Second, DRN privacy may be threatened when the remaining age and local information are used, even if direct identifiers are removed [34-43]. DRN researchers have recognized that there are no satisfactory solutions to privacy risk [43]. Despite such privacy risks, few studies have objectively measured these risks as compared to conventional data sharing–based studies [44-46]. To mitigate the possible risk to a DRN, an objective measurement of the privacy risk should be performed.

Thus, this study aimed to quantify DRN privacy risk by comparing different deidentification levels focusing on personal health identifiers (PHIs) and quasi-identifiers (QIs) of patient information. The key research questions in this study are as follows: (1) What PHIs and QIs are included in a DRN, and how many exist? (2) Using a PHI and QI, when comparing the deidentification level of a CDM to a safe harbor policy, how much will be the decrease in the DRN privacy risk? and (3) What is the true privacy risk of the PHI or QI itself when adjusted for the number of records?

## Methods

### Data Sources

We used the Synthetic Public Use File 5 Percent (SynPUF5PCT) data set, which is a sample data set of the OMOP CDM. The OMOP CDM (version 5.2.2), which was developed by OHDSI [18,47], is a database of relational schema and consists of 37 tables with demographic information, disease natural history, health care cost, etc [48]. The SynPUF5PCT is a synthetic data set with 5% random sampling from a synthetic public use file of the Centers for Medicare and Medicaid Services [49] and complies with the limited data set policy of the HIPAA [32]. The SynPUF5PCT consists of 33 of 37 OMOP CDM tables and is provided from the OHDSI [50]. We used only 12 tables with patient information without missing and null variables from the SynPUF5PCT [51].

### Target PHIs and QIs

**Figure 1:**
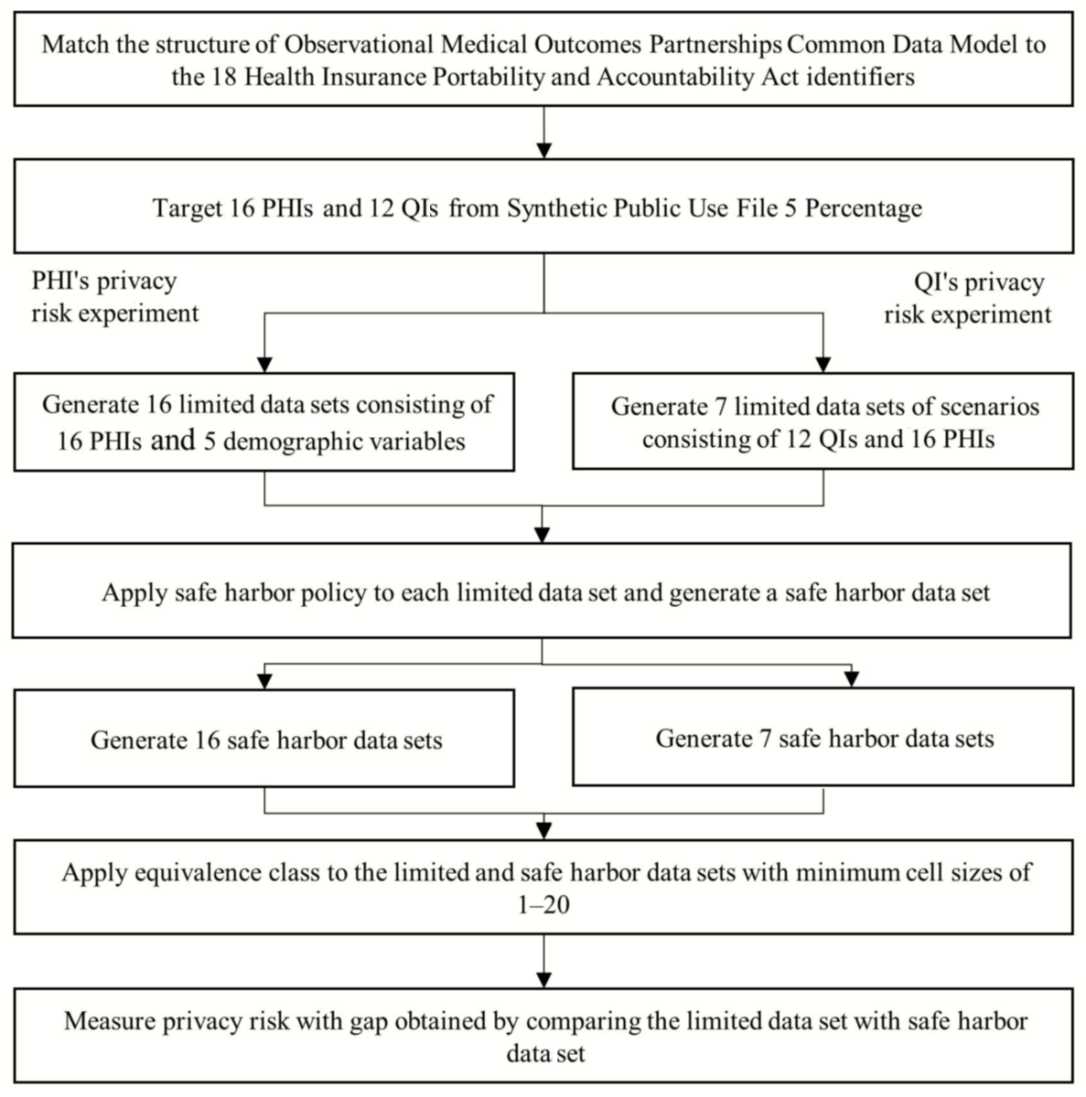
Study workflow. PHI: personal health identifier; QI: quasi-identifier.

In this study, PHIs and QIs were focused on as privacy-threatening patient information by referencing previous studies [52-54]. For the PHIs, we manually matched the structure of the OMOP CDM based on 18 HIPAA identifiers (Figure 1) [55]. For the QIs, we selected the target range in demographic variables (eg, year of birth and gender) and clinical variables (eg, clinical order code) based on previous studies on the privacy risk of QIs [52-54,56,57]. In the 18 HIPAA identifiers, however, dates (excluding the year) and zip codes are defined as PHIs with a QI characteristic [56]. We prioritized the 18 HIPAA identifiers and fixed the dates and zip codes as PHIs instead of QIs. Forty-five PHIs and 17 QIs were detected from the OMOP CDM structure (Multimedia Appendix 1) [58]. Because there were missing tables in the SynPUF5PCT compared to the OMOP CDM, 16 PHIs and 12 QIs were targeted from the SynPUF5PCT (Figure 1 and Table 1). Detailed information for the 28 targeted variables is presented in Multimedia Appendix 2.

**Table 1:** Sixteen personal health identifiers and 12 quasi-identifiers targeted in the Observational Medical Outcomes Partnership common data model based on not null values in the Synthetic Public Use File 5 Percent data set.

Standard clinical tables in the OMOP^a^ CDM^b^	Variable of personal health identifier	Demographic variable of quasi-identifier	Clinical variable of quasi-identifier
Person	Month_of_birth and Day_of_birth	Year_of_birth, Gender_concept_id, Race_concept_id, and Ethnicity_concept_id	N/A^c^
Death	Death_date	N/A	N/A
Device_exposure	Device_exposure_start_date and Device_exposure_end_date	N/A	Device_concept_id
Drug_exposure	Drug_exposure_start_date and Drug_exposure_end_date	N/A	Drug_concept_id
Location	County	State	N/A
Measurement	Measurement_date	N/A	Measurement_concept_id
Observation	Observation_date	N/A	Observation_concept_id
Procedure_occurrence	Procedure_date	N/A	Procedure_concept_id
Visit_occurrence	Visit_start_date and Visit_end_date	N/A	N/A
Condition_occurrence	Condition_start_date and Condition_end_date	N/A	Condition_concept_id
Provider	NPI^d^	N/A	N/A
Care_site	N/A	N/A	Place_of_service_concept_id

^a^OMOP: Observational Medical Outcomes Partnership.

^b^CDM: common data model.

^c^N/A: not applicable.

^d^NPI: national provider identifier.

### Study Design

We conducted privacy risk experiments of the PHIs and QIs. We generated data sets for each experiment. The workflow for this study is shown in Figure 1. In the privacy risk experiment of the PHIs, 16 limited data sets were generated, with each comprising one of the 16 PHIs merged with five common demographic variables (Year_of_birth, Gender_concept_id, Race_concept_id, Ethnicity_concept_id, and State), as in previous clinical studies [53,54]. For example, Condition_start_date, which is the name of data set 1 of the 16 limited data sets, consists of one PHI (Condition_start_date variable) and five common demographic variables. Another example is the Procedure_date data set consisting of one PHI (Procedure_date variable) and five common demographic variables. Thus, each limited data set consists of six variables.

In the QI privacy risk experiment, we mocked up seven scenarios based on the core tables of the OMOP CDM [16,59-61], which are frequently used in the real world. The seven scenarios are as follows: (1) diagnosis, (2) procedure, (3) drug treatment, (4) lab test, (5) device treatment, (6) death, and (7) medical history (Multimedia Appendix 3). Based on the scenarios, seven limited data sets were generated: 10 PHIs and seven QIs were assigned according to the characteristics of each scenario differently, and five demographic variables and six PHIs were used as common variables (Multimedia Appendix 3). For example, the diagnosis scenario consisted of 14 variables as follows: two PHIs (Condition_start_date and Condition_end_date) and one QI (Condition_concept_id), which followed the characteristics of the diagnosis scenario, and 11 common variables were merged.

To compare different deidentification levels for the same data set, we applied the safe harbor policy to the 16 limited data sets. For example, when the safe harbor policy was applied to the limited data set, the PHIs were partially or completely masked. The date type (such as start date, end date, and death date) was masked from “YYYY-MM-DD” to “YYYY-**-**.” In other words, they used only the “year”. The others (such as Month_of_birth, Day_of_birth, NPI, and County) were completely masked. We additionally generated 16 and seven safe harbor data sets for PHIs and QIs, respectively, by applying the safe harbor policy on the limited data sets.

### Privacy Risk Evaluation Metrics

An equivalence class (EC) denotes a group of indistinguishable record forms with common attributes. The common attribute sizes that are included in each group can be represented as the calculated size of the EC [46]. An EC size of one represents the highest possibility of privacy disclosure for a certain patient’s information [56]. In contrast, if the size is maximum, it indicates the highest deidentification level of the data set. In previous studies, the minimum cell size was an empirically defined threshold with the calculated EC size [56,57]. The minimum cell size determines the level of deidentification and measures the privacy risk in the data set. The most commonly used minimum cell size in practice is five, and a larger size, such as 20, is used for data sets that include highly sensitive patient information [56]. The minimum cell size, calculated by the EC, was compared for both the limited and safe harbor data sets.

The trust differential mechanism represents the privacy risk of a data set with a gap obtained by comparing two different deidentification levels [54]. The gap represents the following two factors: (1) the quantified difference of the deidentification level and (2) the degree of decrease in privacy risk. In other words, when a certain privacy policy applies to the data set that complies with another privacy policy, a gap will occur between the two different privacy policies, which have different deidentification levels. Therefore, the gap indicates that the data set’s privacy level with the lower deidentification privacy policy could be protected as the difference that arises when the higher privacy policy is applied.

Through the PHI and QI privacy risk experiments, we measured privacy risk in terms of the following two aspects: (1) measurements based on the number of total records in each data set and (2) measurements based on the identical number of records through random sampling from each data set. In the first aspect, we considered that clinical studies perform analysis with clinical tables according to clinical scenarios [16,59-61]; thus, we measured privacy risk with the number of total records in the data set generated by referring to previous studies [53,54]. With the number of total records, we compared the limited and safe harbor data sets based on the total records of each PHI and QI. Then, we measured with different minimum cell sizes from each PHI and QI experiment. To measure PHI privacy risk, we compared the limited and safe harbor data sets with the maximum EC size and a minimum cell size of one. In the QI privacy risk experiment, we compared the limited and safe harbor data sets with a minimum cell size of 1 to 20. In the second aspect, we extracted 1000, 10,000, 100,000, and 1 million random samples from each limited and safe harbor data set and iterated them 100 times. With the iterated random samples, we calculated the average of the minimum cell size 1 and then compared the limited and safe harbor data sets for PHIs and QIs.

## Results

### Overview

Overall, when compared with the limited and safe harbor data sets, privacy risk was reduced in both PHIs and QIs according to the trust differential gap. For the trust differential gap of a minimum cell size of one, there are two overall results. In the number of total records, the trust differential gaps of PHIs and QIs averaged 31.448% and 73.798%, respectively. In the random samples, the trust differential gaps of PHIs and QIs averaged 18.869% and 6.493% (1000 samples), 50.730% and 33.248% (10,000 samples), 74.013% and 60.306% (100,000 samples), and 50.744% and 71.868% (1,000,000 samples), respectively (Table 2).

**Table 2:** The averaged trust differential gap according to total records and random samples.

Number of total records^a^ and sample^b^		Trust differential gap^c^ with a minimum cell size of one^d^	
		Personal health identifier (mean percentage)	Quasi-identifier (mean percentage)
Number of total records		31.448%	73.798%
**Sample**			
	1000	18.869%	6.493%
	10,000	50.730%	33.248%
	100,000	74.013%	60.306%
	1,000,000	50.744%	71.868%

^a^Number of total records is each personal health identifier’s total record.

^b^Sample is the number of random samples (ie, 1000, 10,000, 100,000, or 1 million) from the limited and safe harbor data sets.

^c^Trust differential gap is the difference obtained by comparing two data sets to measure privacy risk.

^d^Minimum cell size of one is the percentage of unique records. This can be expressed with the number of unique records as the numerator and the number of total records as the denominator.

### Evaluation of the Personal Health Identifier Privacy Risk of the DRN

In the number of total record results of the limited data set, the variable with the most included minimum cell size of one was Death_date, which was 98.787% (1141/1155). In addition, the maximum EC size of two for Death_date means that every record consists of only two value types. In Death_date of the safe harbor data set, the minimum cell size of one was 87.359% (1009/1155), and the maximum EC size was three. Even though the safe harbor policy was applied, privacy was still threatened. In the Death_date trust differential gap, the gap with a minimum cell size of one was 11.428%, and the maximum EC size was one. The maximum EC size of one is the lowest trust differential gap among all the maximum EC size gaps. In the limited data set, the variable with the least minimum cell size of one was Condition_end_date, which was 4.540% (146,727/3,231,730). In Condition_end_date from the safe harbor data set, the minimum cell size of one was 0.003% (125/3,231,730). Even though the safe harbor policy was applied, the records of a minimum cell size of one did not significantly decrease. In the Condition_end_date trust differential gap, the minimum cell size of one was 4.536%, and the maximum EC size was 2348. This maximum EC size of 2348 was the highest trust differential gap among all the maximum EC size gaps. In the trust differential gaps with a minimum cell size of one, the NPI variable had the highest trust differential gap of 71.236%, which was the difference between the limited (71.244%) and safe harbor (0.007%) data sets. For Drug_exposure_start_date and Drug_exposure_end_date, both data sets exhibited the same maximum EC size and a minimum cell size of one.

Day_of_birth consists of the day part of the date of birth and was already deidentified as “1” in the SynPUF5PCT data set (eg, “dd” to “1”); thus, every patient had the exact same Day_of_birth value. Because it was the same deidentified method as for the safe harbor policy, the Day_of_birth trust differential gap was zero (Table 3). It could be provided as a statistical baseline for five demographic variables without any PHI variables. When the measured result of the Day_of_birth variable (13.079%) was compared with that of the Condition_end_date variable, the result of the Condition_end_date variable was lower by 8.539 percentage points (from 13.079% to 4.540%), and when it was compared with that of the Death_date variable, the result of the Death_date variable was higher by 85.708 percentage points (from 13.079% to 98.787%).

**Table 3:** Comparison of 16 personal health identifier variables and five demographic variables of the SynPUF5PCT with limited and safe harbor data sets in terms of a minimum cell size of one and the maximum size of the equivalence class.

Variable^a^	Number of total records^b^	Limited data set				Safe harbor data set				Trust differential gap^c^	
		Number of unique records^d^	Minimum cell size of one^e^ (%)	Maximum size of the equivalence class^f^	Number of unique records^d^		Minimum cell size of one^e^ (%)	Maximum size of the equivalence class^f^	Minimum cell size of one (%)		Maximum size of the equivalence class
Visit_start_date	1,218,881	771,684	63.310	10	581		0.047	888	62.952		878
Visit_end_date	1,218,881	771,891	63.327	10	581		0.0395	889	62.960		879
Death_date	1155	1141	98.787	2	1009		87.359	3	11.428		1
Condition_start_date	3,231,730	146,828	4.543	45	137		0.004	2391	4.538		2346
Condition_end_date	3,231,730	146,727	4.540	45	125		0.003	2393	4.536		2348
Procedure_date	3,024,452	257,161	8.502	64	201		0.006	2180	8.495		2116
Measurement_date	741,161	168,180	22.691	43	595		0.080	575	22.610		532
Observation_date	420,986	182,497	43.349	28	983		0.233	335	43.115		307
Device_exposure_start_date	47,655	13,232	27.766	40	3190		6.693	218	21.073		178
Device_exposure_end_date	47,655	13,219	27.739	40	3191		6.696	187	21.043		147
Drug_exposure_start_date	158,316	55,042	34.767	45	2845		1.797	409	32.970		364
Drug_exposure_end_date	158,316	55,042	34.767	45	2845		1.797	409	32.970		364
Month_of_birth	25,200	14,508	57.571	8	3296		13.079	49	44.492		41
Day_of_birth	25,200	3296	13.079	49	3296		13.079	49	0		0
NPI^g^	1,215,317	865,840	71.244	70	91		0.007	2247	71.236		2177
County	25,200	18,103	71.837	12	3296		13.079	49	58.757		37
Average	N/A^h^	N/A	40.488	34.75	N/A		8.999	829.437	31.448		771.937

^a^Variable refers to the variable targeted from the Observational Medical Outcomes Partnership common data model as the personal health identifier.

^b^Number of total records is each personal health identifier’s total record.

^c^Trust differential gap is the difference obtained by comparing two data sets to measure privacy risk.

^d^Number of unique records is the number of records with a common attribute size of one within the total record.

^e^Minimum cell size of one is the percentage of unique records. This can be expressed with the number of unique records as the numerator and the number of total records as the denominator.

^f^Maximum size of the equivalence class is the largest size of the indistinguishable common attributes.

^g^NPI: national provider identifier.

^h^N/A: not applicable.

In randomly sampled PHIs, privacy risk reduction was different depending on the number of samples (Table 4 and Multimedia Appendix 4). The variables with a highly ranked trust differential gap were Device_exposure_start_date (1000 samples) (33.730%; 87.705% and 53.975% in the limited and safe harbor data sets, respectively), NPI (10,000 samples) (83.852%; 98.945% and 15.094% in the limited and safe harbor data sets, respectively), Visit_start_date (100,000 samples) (92.566%; 95.583% and 3.016% in the limited and safe harbor data sets, respectively), and NPI (1,000,000 samples) (73.588%; 73.599% and 0.011% in the limited and safe harbor data sets, respectively).

Overall, for 1000 random samples, both data sets consisted primarily of the minimum cell size of one. In the limited data set, the variables with the most and fewest included minimum cell size of one records were Visit_end_date (99.978%) and Day_of_birth (73.754%), respectively. In the safe harbor data set, the variables with the most and fewest included minimum cell size of one records were Death_date (89.044%) and NPI (67.377%), respectively (Table 4). For Visit_end_date in the limited data set with the most included minimum cell size of one records, after applying the safe harbor policy, the minimum cell size of one records of the Visit_end_date variable decreased to 86.171% (861.710/1000). Even though the safe harbor policy was applied, the minimum cell size of one records did not decrease significantly. Death_date, with the most included minimum cell size of one records in the safe harbor data set, had a trust differential gap of 9.862% (98.906% and 89.044% in the limited and safe harbor data sets, respectively). The privacy risk did not decrease significantly after applying the safe harbor policy. In the trust differential gap, the variable with the highest gap was Device_exposure_start_date (33.730%; 87.705% and 53.975% in the limited and safe harbor data sets, respectively). When the safe harbor policy was applied, the Death_date privacy risk could be significantly reduced. In the number of total records of the limited and safe harbor data sets, with a minimum cell size of one, the most privacy-threatening variables were Death_date (98.787%) and Death_date (87.359%), respectively. However, in the random sample of 1000, it was Visit_end_date (99.978%) and Death_date (89.044%), respectively. Therefore, we verified that privacy-threatening variables could differ depending on the number of records. Detailed random sampled results are displayed in Multimedia Appendix 4.

**Table 4:** Comparison of records with a minimum cell size of one between the limited and safe harbor data sets from 16 personal health identifier data sets.

Sample^a^ and variable^b^			Limited data set					Safe harbor data set					Trust differential gap^c^ (%)		
			Number of minimum cell sizes of one^d^					Number of minimum cell sizes of one^d^							
			Mean^e^ (SD^f^)		Percentage^g^ (%)		Mean^e^ (SD^f^)			Percentage^g^ (%)					
**1000 samples**															
	Visit_start_date	999.26 (1.125)		99.926		859.68 (16.229)			85.968		13.958				
	Visit_end_date	999.78 (0.629)		99.978		861.71 (15.086)			86.171		13.807				
	Death_date	989.06 (2.155)		98.906		890.44 (7.478)			89.044		9.862				
	Condition_start_date	998.44 (1.766)		99.844		857.59 (15.178)			85.759		14.085				
	Condition_end_date	998.12 (2.006)		99.812		858.7 (16.45)			85.87		13.942				
	Procedure_date	998.24 (1.804)		99.824		858.61 (16.504)			85.861		13.963				
	Measurement_date	995.14 (2.971)		99.514		855.11 (15.321)			85.511		14.003				
	Observation_date	997.54 (2.162)		99.754		854.79 (14.238)			85.479		14.275				
	Device_exposure_start_date	877.05 (13.107)		87.705		539.75 (16.877)			53.975		33.73				
	Device_exposure_end_date	875.34 (16.05)		87.534		539.68 (20.112)			53.968		33.566				
	Drug_exposure_start_date	956.34 (8.669)		95.634		720.7 (17.729)			72.07		23.564				
	Drug_exposure_end_date	956.34 (8.669)		95.634		720.7 (17.729)			72.07		23.564				
	Month_of_birth	971.47 (7.612)		97.147		738.4 (17.707)			73.84		23.307				
	Day_of_birth	737.54 (17.774)		73.754		737.54 (17.774)			73.754		0				
	NPI^h^	998.8 (1.775)		99.88		673.77 (19.212)			67.377		32.503				
	County	979.69 (6.59)		97.969		738.46 (16.856)			73.846		24.123				
	Average	N/A^i^		N/A		N/A			N/A		18.869				

^a^Sample is the number of random samples (ie, 1000, 10,000, 100,000, or 1 million) from the limited and safe harbor data sets.

^b^Variable is the variable targeted from the Observational Medical Outcomes Partnership common data model as the personal health identifier.

^c^Trust differential gap is the difference obtained by comparing two data sets to measure privacy risk.

^d^Number of minimum cell sizes of one is the number of records with a unique record among the total records.

^e^Mean is the average of the quantity with a minimum cell size of one obtained by iterating the random sampling of each variable 100 times.

^f^SD is the standard deviation of the quantity with a minimum cell size of one obtained by iterating random sampling of each variable 100 times.

^g^Percentage is the percentage of the quantity with a minimum cell size of one. The numerator is the mean of the minimum cell size of one, which was obtained from 100 iterations, and the denominator was the number of random samples.

^h^NPI: national provider identifier.

^i^N/A: not applicable.

### Evaluation of the Quasi-Identifier Privacy Risk of the DRN

**Figure 2:**
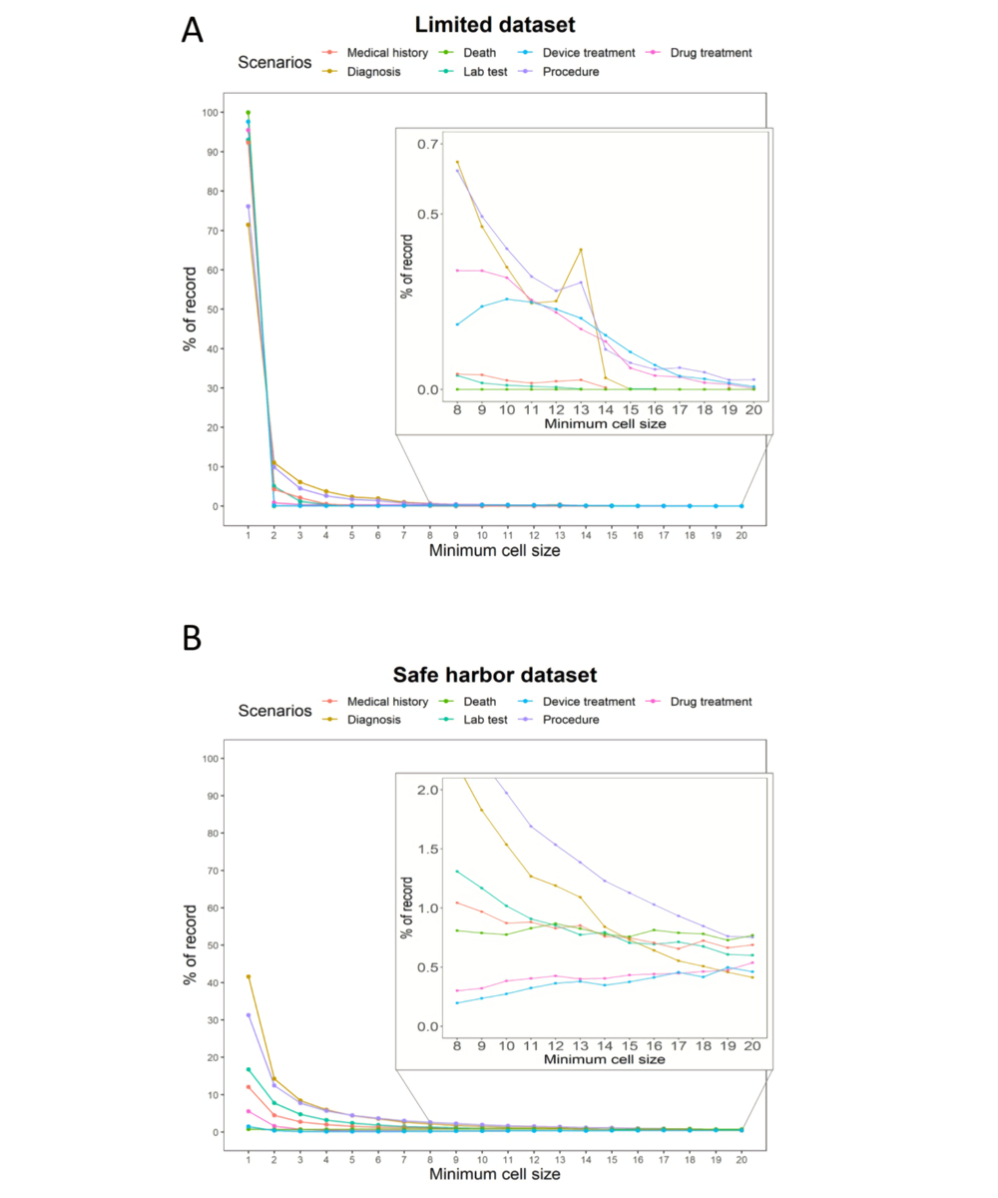
Percentage of records measuring the quasi-identifier privacy risk with a minimum cell size of 1–20 for the (A) limited and (B) safe harbor data sets. The flattened lines are expanded (inner graph).

In the results for the number of total records, the privacy risk of the QI with a minimum cell size of 1 to 20 was measured in the limited and safe harbor data sets. As shown in Figure 2, for the minimum cell size of one, the minimum and maximum percentages in the seven scenarios were 71% and 99%, respectively, in the limited data set (Figure 2A) and 0.7% and 41%, respectively, in the safe harbor data set (Figure 2B). The QI privacy risk was represented with a minimum cell size of one to five (Multimedia Appendix 5 and Table 5). For the minimum cell size of one in the limited data set, the Diagnosis (71.465%) and Procedure (76.123%) scenarios showed lower privacy risks than the other five scenarios (Drug treatment [95.475%], Lab test [93.012%], Medical history [92.353%], Death [99.997%], and Device treatment [97.647%]). For the Death scenario, the limited data set records were concentrated in the minimum cell size of one to two. The average gaps between the limited and safe harbor data sets, with the minimum cell size of one to five decreased from 73.798% to 54.548%. For the gaps of the minimum cell size of one, the Diagnosis scenario showed the smallest gap (28.869%), whereas the Death scenario showed the largest gap (99.212%).

**Table 5:** Percentage of records measuring quasi-identifier privacy risk with gaps between the limited and safe harbor data sets with a minimum cell size of one, two, and five.

Scenarios	Number of total records^a^	Limited data set				Safe harbor data set				Trust differential gap^b^		
		Minimum cell size of one, two, and five, percentage^c^ (record^d^)				Minimum cell size of one, two, and five, percentage^c^ (record^d^)				Minimum cell size of one, two, and five, percentage^c^		
		1	2	5	1		2	5	1		2	5
Diagnosis	3,369,468	71.465 (2,407,996)	11.049 (186,162)	2.333 (15,726)	41.595 (1,401,556)		14.248 (240,043)	4.412 (29,737)	29.869		35.361	19.986
Procedure	3,105,665	76.123 (2,364,135)	9.902 (153,767)	1.731 (10,752)	31.251 (970,568)		12.472 (193,672)	4.460 (27,708)	44.871		42.301	33.173
Drug treatment	1,300,649	95.475 (1,241,796)	0.895 (5826)	0.306 (796)	5.558 (72,292)		1.625 (10,569)	0.356 (927)	89.917		89.187	88.611
Lab test	1,622,884	93.012 (1,509,486)	5.043 (40,923)	0.138 (448)	16.749 (271,819)		7.748 (62,875)	2.385 (7744)	76.263		73.558	64.958
Medical history	1,348,569	92.353 (1,245,455)	4.286 (28,900)	0.224 (606)	12.079 (162,898)		4.466 (30,115)	1.550 (4183)	80.274		80.094	76.759
Death	1,218,881	99.997 (1,218,845)	0.003 (18)	0 (0)	0.784 (9557)		0.686 (4185)	0.719 (1755)	99.212		98.529	0
Device treatment	1,247,726	97.647 (1,218,368)	0.152 (954)	0.059 (149)	1.464 (18,271)		0.380 (2372)	0.139 (348)	96.182		95.955	95.625
Average	N/A^e^	N/A	N/A	N/A	N/A		N/A	N/A	73.798		73.569	54.458

^a^Number of total records denotes each total record of the scenarios.

^b^Trust differential gap indicates the differences obtained by comparing two data sets to measure privacy risk.

^c^Minimum cell size of one, two, and five represents the percentage of records that have a common attribute size of one, two, and five, respectively. This percentage is presented as the records of minimum cell size of one, two, and five as the numerator and the total number of records as the denominator.

^d^Record is the number of records with a common attribute size of one, two, and five within the total records.

^e^N/A: not applicable.

In the random samples with a minimum cell size of one, (1) the average percentage of the limited data set decreased from 99.986% to 99.327%, (2) the average percentage of the safe harbor data set decreased from 93.493% to 21.460%, and (3) the average trust differential gap increased from 6.493% to 71.868% (Table 6). In the limited data set with 1000 to 1 million random samples, the scenario with the most included records of a minimum cell size of one was the Death scenario (1000 to 100,000 random samples had 99.999% and 1 million had 99.998%). In the safe harbor data set with 1000 to 1 million random samples, the scenario with the most included records of a minimum cell size of one was the Diagnosis scenario (1000 random samples had 99.858%, 10,000 had 98.685%, 100,000 had 89.758%, and 1 million had 60.361%). In the order of the four random samples, the scenarios with the highest trust differential gap were Device_treatment (1000 random samples: 12.980%, 99.980% and 87.000% in the limited and safe harbor data sets, respectively; 10,000 random samples: 60.118%, 99.831% and 39.713% in the limited and safe harbor data sets, respectively; 100,000 random samples: 93.598%, 98.805% and 5.207% in the limited and safe harbor data sets, respectively) and Death (1 million random samples: 99.063%, 99.998% and 0.934% in the limited and safe harbor data sets, respectively). When the safe harbor policy was applied, privacy risks were significantly reduced. In the number of total records, the most privacy-threatening scenarios were Death (99.997%) and Diagnosis (41.595%) in the limited and safe harbor data sets, respectively, with a minimum cell size of one. In the random samples with a minimum cell size of one in the limited data set, the most privacy-threatening scenario was Death, which had privacy risks of 99.999% (1000 to 100,000 random samples) and 99.998% (1 million random samples). In the safe harbor data set, Diagnosis had privacy risks of 99.858% (1000 random samples), 98.685% (10,000 random samples), 89.758% (100,000 random samples), and 60.361% (1 million random samples).

**Table 6:** Comparison of records with a minimum cell size of one between the limited and safe harbor data sets from seven scenarios.

Sample^a^ and scenario^b^			Limited data set					Safe harbor data set					Trust differential gap^c^ (%)		
			Number of minimum cell sizes of one^d^					Number of minimum cell sizes of one^d^							
			Mean^e^ (SD^f^)		Percentage^g^ (%)		Mean^e^ (SD^f^)			Percentage^g^ (%)					
**1000**															
	Diagnosis	999.800		99.980		998.580			99.858		0.122				
	Procedure	999.760		99.976		997.400			99.740		0.236				
	Drug_treatment	999.860		99.986		889.470			88.947		11.039				
	Lab_test	999.900		99.990		956.910			95.691		4.299				
	Medical_history	999.960		99.996		932.320			93.232		6.764				
	Death	999.990		99.999		899.830			89.983		10.016				
	Device_treatment	999.800		99.980		870.000			87.000		12.980				
	Average	N/A^h^		99.986		N/A			93.493		6.493				
**10,000**															
	Diagnosis	9975.850		99.759		9868.540			98.685		1.073				
	Procedure	9974.680		99.747		9743.830			97.438		2.309				
	Drug_treatment	9980.620		99.806		4642.820			46.428		53.378				
	Lab_test	9993.920		99.939		7320.070			73.201		26.739				
	Medical_history	9989.730		99.897		6226.700			62.267		37.630				
	Death	9999.980		99.9990		4851.230			48.512		51.487				
	Device_treatment	9983.140		99.831		3971.310			39.713		60.118				
	Average	N/A		99.854		N/A			66.606		33.248				
**100,000**															
	Diagnosis	97,724.930		97.725		89,757.540			89.758		7.967				
	Procedure	97,742.680		97.743		82,093.250			82.093		15.649				
	Drug_treatment	98,419.410		98.419		12,062.980			12.063		86.356				
	Lab_test	99,375.690		99.376		42,164.130			42.164		57.212				
	Medical_history	99,022.020		99.022		28,552.750			28.553		70.469				
	Death	99,999.640		99.999		9106.630			9.107		90.892				
	Device_treatment	98,804.960		98.805		5206.990			5.207		93.598				
	Average	N/A		98.727		N/A			38.420		60.306				
**1,000,000**															
	Diagnosis	846,819.090		84.682		603,607.950			60.361		24.321				
	Procedure	864,575.710		86.458		472,502.940			47.250		39.207				
	Drug_treatment	957,078.730		95.708		59,825.330			5.983		89.725				
	Lab_test	951,528.090		95.153		206,809.130			20.681		74.472				
	Medical_history	936,158.630		93.616		134,617.450			13.462		80.154				
	Death	999,975.900		99.998		9344.160			0.934		99.063				
	Device_treatment	976,802.140		97.680		15,496.020			1.550		96.131				
	Average	N/A		93.327		N/A			21.460		71.868				

^a^Sample is the number of random samples (ie, 1000, 10,000, 100,000, or 1 million) from the limited and safe harbor data sets.

^b^Scenario is the variable targeted from the Observational Medical Outcomes Partnership common data model as the personal health identifier.

^c^Trust differential gap is the difference obtained by comparing two data sets to measure privacy risk.

^d^Number of minimum cell sizes of one is the number of records with a unique record among the total records.

^e^Mean is the average of the quantity with a minimum cell size of one obtained by iterating the random sampling of each variable 100 times.

^f^SD is the standard deviation of the quantity with a minimum cell size of one obtained by iterating random sampling of each variable 100 times.

^g^Percent is the percentage of the quantity with a minimum cell size of one. The numerator is the mean of the minimum cell size of one, which was obtained from 100 iterations, and the denominator was the number of random samples.

^h^N/A: not applicable.

## Discussion

### Principal Findings

In this study, we quantified the DRN privacy risk focusing on PHIs and QIs using 18 HIPAA identifiers and the findings of previous studies [34-43]. To measure the DRN privacy risk, we compared the limited data set, consisting of PHIs and QIs from the SynPUF5PCT data set, with the safe harbor data set generated by applying the safe harbor policy on the limited data set. More specifically, privacy risk was measured with the gap obtained between the two data sets, based on the trust differential, applying the threshold of the minimum cell size with the calculated size by the EC. We verified that the PHIs and QIs increased the DRN privacy risk. However, the privacy risk decreased overall when the safe harbor policy was applied to the DRN. To the best of our knowledge, this is the first study to verify that PHIs and QIs may threaten patient privacy within DRNs.

Prior studies have shown that patient privacy is threatened by PHIs and QIs within clinical databases [53,54]. The DRN of this study may have the same privacy risk as those in previous studies because the DRN at a single site follows a conventional database, although it does not share data [34-41]. Therefore, the privacy risk in a DRN should be quantified and objectively measured for three important reasons. First, because existing patient information in a CDM affects the privacy risk, the DRN privacy risk can be mitigated by providing objectively measured PHI and QI privacy risks [62]. Second, researchers can understand the mechanism of privacy risk change with the objective differences measured by comparing two different deidentification levels of data sets [63]. Finally, an objective measurement of privacy risk will contribute to the design of more secure privacy protection methods suitable for a DRN.

### Consideration for Measuring Privacy Risk From Variable Characteristics

The PHI results, which measure the privacy risk, were verified in two different deidentification levels and indicated a much greater privacy risk reduction in the safe harbor data set than in the limited data set. In addition, we found that privacy risks differ depending on PHI characteristics. The privacy risk of the Visit_start_date variable, which occurs multiple times per patient, was significantly reduced after applying the safe harbor policy. However, the Death_date variable, which occurs only once per patient, still had many remaining unique records after the safe harbor policy was applied. The State variable, which is one of the demographic variables in the data set of the Death_date variable, still had unique values because it had not been deidentified by the safe harbor policy. Although the NPI variable had the highest reduction rate of privacy risk after applying the safe harbor policy, we found that it could not be used as data because it was completely masked. For the Day_of_birth as a statistical baseline, we compared the Day_of_birth with other PHI variables and could interpret a privacy risk according to the characteristics of the variable as follows. First, because each patient had multiple points for the Condition_end_date value in the SynPUF5PCT, there were fewer unique records relatively. Thus, the privacy risk of Condition_end_date was lower than that of Day_of_birth. Second, because every patient had only one point for the Death_date value, most of them had unique records. Thus, the privacy risk of Death_date was higher than that of Day_of_birth.

In the results of QI, when the limited data set had a minimum cell size of one, the privacy risk differed based on the characteristics of the scenario. In our study, we found that the QI privacy risks of the Drug treatment, Lab test, Medical history, Death, and Device treatment scenarios decreased on average 1.3 times more than those of the Diagnosis and Procedure scenarios, with a minimum cell size of one. The reason for the relatively low reduction in privacy risk under the Diagnosis and Procedure scenarios is that clinical order codes, such as Condition_concept_id and Procedure_concept_id, which used QIs, were prescribed three times on average with the same code.

The privacy risk could differ depending on the characteristics of variables, and the “balls and bins problem” theoretical basis supports our research [64]. As the number of bins increases, it could frequently take only one ball to fill than fewer bins. Similarly, the Visit_end_date variable, with 1096 distinct values (“bins”), consisted of more unique records (“only one ball”) than the Month_of_birth with 12 distinct values. Consequently, a privacy protection approach must be customized or optimized by considering the characteristics of each variable.

### Consideration for Measuring Privacy Risk From Record Extraction

Through the random samples, we found the following two facts: (1) Depending on the number of records, the privacy-threatening variable or scenario could differ and (2) The influence of safe harbor policy could differ depending on the number of records, because the number of unique records, which are included with PHI data sets or QI scenarios, differs according to each random sampling. Therefore, to measure the true privacy risk of PHIs and QIs, it is necessary to compare the same records through random sampling.

A minimum cell size of five, which has been a commonly used threshold in previous studies [56], may be difficult to apply as a threshold for measuring the DRN privacy risk. In the QI privacy risk experiment, the Death scenario of the limited data set was not appropriate for a minimum cell size of five because the records were concentrated in a minimum cell size of one to two. Therefore, our results reflect the fact that a minimum cell size of five may not be suitable for the current DRN. However, it should be recognized that the captured features may differ according to the data set used. Therefore, further research is required using various real-world data sets to find an appropriate minimum cell size that can contribute to the measurement of the DRN privacy risk.

### Limitations

This study has some limitations. First, this study used a public data set (SynPUF5PCT), which does not handle all PHIs or QIs existing in a DRN. Therefore, we could not consider the CDM of real-world data sets generated by each institution. However, the results of this study are reliable because the SynPUF5PCT data set is an officially published data set by the OHDSI [50]. Second, when measuring the QI privacy risk, some QIs were considered based on scenarios and not based on all variables. Thus, we did not handle the privacy risk considering the combination of all QIs. However, the CDM does not use all variables because the research is based on clinical questions [59]. In addition, we focused on the frequently used scenarios. Third, we did not consider some PHIs and QIs within free text from Note and Note_nlp tables [48], because in our research methodology, PHIs and QIs are detected in the structure of OMOP CDM based on 18 HIPAA identifiers and not in the free text. However, previous studies have indicated that free text includes not only PHIs and QIs but also direct identifiers [65,66]. Therefore, further research needs to include a free text data set. Fourth, we did not consider privacy risk depending on the timespan. Because the SynPUF5PCT data set used in this study contained only 3-year records (2008-2010) and the Day_of_birth variable had already been deidentified as “1,” we could not measure privacy risk according to an extended (such as 20-year records) or a narrowed (such as single-week records) timespan. A future study should consider timespan-related privacy.

### Conclusions

In this study, we validated and quantified the privacy risks of PHIs and QIs in the DRN. We objectively measured the privacy risk reduction with the gaps obtained by comparing a safe harbor policy with the DRN. In addition, we measured the true privacy risk of PHIs and QIs by random sampling to adjust for the influence of the number of records. Therefore, it is necessary to reinforce a level of privacy protection for each institution because the DRN involves big data research based on multi-institution collaboration. Our study findings can help in constructing an advanced DRN environment that protects these privacy risks as a quality measurement index.

## Appendix

Multimedia Appendix 1 Forty-five personal health identifiers and 17 quasi-identifiers in the structure of the Observational Medical Outcome Partnership common data model.

Multimedia Appendix 2 Detailed information of 16 personal health identifier variables and 12 quasi-identifier scenarios.

Multimedia Appendix 3 Seven scenarios with personal health identifiers and quasi-identifiers in the Observational Medical Outcome Partnership common data model, based on not null values in the Synthetic Public Use File 5 Percent data set.

Multimedia Appendix 4 Random sampling and 100 iterations were conducted to compare records with a minimum cell size of one between the limited and safe harbor data sets from 16 personal health identifier data sets.

Multimedia Appendix 5 Percentage of records measuring quasi-identifier privacy risk with gaps between the limited and safe harbor data sets with a minimum cell size of one to five.

## Abbreviations

CDM: common data model
DRN: distributed research network
EC: equivalence class
HIPAA: Health Insurance Portability and Accountability Act
NPI: national provider identifier
OHDSI: Observational Health Data Sciences and Informatics
OMOP: Observational Medical Outcome Partnership
PHI: personal health identifier
QI: quasi-identifier
SynPUF5PCT: Synthetic Public Use File 5 Percent
