# Supplementary material for: Evaluation of the Privacy Risks of Personal Health Identifiers and Quasi-Identifiers in a Distributed Research Network: Development and Validation Study
Source: JMIR Med Inform. 2021 May 31;9(5):e24940. doi: 10.2196/24940 (PMC8204238; doi:10.2196/24940)
Supplement: Multimedia Appendix 1 [file medinform_v9i5e24940_app1.docx]

Multimedia Appendix 1. Forty-five personal health identifiers and 17 quasi-identifiers in the structure of the Observational Medical Outcome Partnership common data model.

| Standard clinical tables in OMOP CDM | Personal Health Identifier | Demographic variable of Quasi-Identifier | Clinical Variable of Quasi-Identifier |
| --- | --- | --- | --- |
| Person | Month_of_birth, Day_of_birth, Birth_datetime | Year_of_birth, Gender_concept_id, Race_concept_id, Ethnicity_concept_id |  |
| Specimen | Specimen_date, Specimen_datetime, Specimen_source_id, |  | Specimen_concept_id |
| Death | Death_date, Death_datetime |  |  |
| Device_exposure | Device_exposure_start_date, Device_exposure_start_datetime, Device_exposure_end_date, Device_exposure_end_datetime |  | Device_concept_id |
| Drug_exposure | Drug_exposure_start_date, Drug_exposure_start_datetime, Drug_exposure_end_date, Drug_exposure_end_datetime, verbatim_end_date |  | Drug_concept_id |
| Location | Address_1, Address_2, City, Zip, County | State |  |
| Measurement | Measurement_date, Measurement_datetime |  | Measurement_concept_id |
| Observation | Observation_date, Observation_datetime |  | Observation_concept_id |
| Procedure_occurrence | Procedure_date, Procedure_datetime |  | Procedure_concept_id |
| Visit_occurrence | Visit_start_date, Visit_start_datetime, Visit_end_date, Visit_end_datetime |  |  |
| Condition_occurrence | Condition_start_date, Condition_start_datetime,  Condition_end_date, Condition_end_datetime |  | Condition_concept_id |
| Provider | Provider_name, NPI, Dea | Year_of_birth, Gender_concept_id | Specialty_concept_id |
| Care_site |  |  | Place_of_service_concept_id |
| Payer_plan_period | Payer_plan_period_start_date, Payer_plan_period_end_date |  |  |
| Note | Note_date, Note_datetime |  | Note_class_concept_id |
| Note_Nlp | Nlp_date, Nlp_date_time |  |  |
