# Supplementary material for: Evaluation of the Privacy Risks of Personal Health Identifiers and Quasi-Identifiers in a Distributed Research Network: Development and Validation Study
Source: JMIR Med Inform. 2021 May 31;9(5):e24940. doi: 10.2196/24940 (PMC8204238; doi:10.2196/24940)
Supplement: Multimedia Appendix 2 [file medinform_v9i5e24940_app2.docx]

Multimedia Appendix 2. Detailed information of 16 personal health identifier variables and 12 quasi-identifier scenarios.

| Variable | | | | | | | | Number of total record^a^ | Distinct variable^b^ | | Summary | | | | |
| --- | --- | --- | --- | --- | --- | --- | --- | --- | --- | --- | --- | --- | --- | --- | --- |
| Continuous variable | | | | | | | |  | Count^c^ | Percent^d^ (%) | min | 1^st^ quartile  (Q1=25%) | median | 3^rd^ quartile (Q3=75%) | max |
|  | | Personal Health Identifier | Month_of_birth | | | | | 25 200 | 12 | 0.048 | 1 | 3 | 6 | 9 | 12 |
|  |  |  | Death_date | | | | | 1 155 | 36 | 3.117 | 2008-01-01 | 2008-10-01 | 2009-07-01 | 2010-04-01 | 2010-12-01 |
|  |  |  | Device_exposure_start_date | | | | | 47 655 | 1 116 | 2.342 | 2007-12-12 | 2008-09-29 | 2009-05-06 | 2009-12-11 | 2010-12-31 |
|  |  |  | Device_exposure_end_date | | | | | 47 655 | 1 112 | 2.333 | 2008-01-01 | 2008-10-12 | 2009-05-20 | 2009-12-24 | 2010-12-31 |
|  |  |  | Drug_exposure_start_date | | | | | 158 316 | 1 112 | 0.702 | 2007-12-12 | 2008-09-21 | 2009-05-10 | 2009-12-30 | 2010-12-31 |
|  |  |  | Drug_exposure_end_date | | | | | 158 316 | 1 206 | 0.762 | 2007-12-12 | 2008-10-21 | 2009-06-09 | 2010-01-30 | 2011-03-31 |
|  |  |  | Measurement_date | | | | | 741 161 | 1 109 | 0.150 | 2007-12-12 | 2008-09-20 | 2009-05-01 | 2009-12-18 | 2010-12-31 |
|  |  |  | Observation_date | | | | | 420 986 | 1 112 | 0.264 | 2007-11-27 | 2008-10-19 | 2009-06-04 | 2010-01-13 | 2010-12-31 |
|  |  |  | Procedure_date | | | | | 3 024 452 | 1 118 | 0.037 | 2007-11-27 | 2008-09-20 | 2009-05-03 | 2009-12-21 | 2010-12-31 |
|  |  |  | Visit_start_date | | | | | 1 218 881 | 1 118 | 0.092 | 2007-11-27 | 2008-09-21 | 2009-05-04 | 2009-12-23 | 2010-12-31 |
|  |  |  | Visit_end_date | | | | | 1 218 881 | 1 096 | 0.090 | 2008-01-01 | 2008-09-21 | 2009-05-05 | 2009-12-24 | 2010-12-31 |
|  |  |  | Condition_start_date | | | | | 3 231 730 | 1 118 | 0.035 | 2007-11-27 | 2008-09-24 | 2009-05-09 | 2009-12-28 | 2010-12-31 |
|  |  |  | Condition_end_date | | | | | 3 231 730 | 1 096 | 0.034 | 2008-01-01 | 2008-09-24 | 2009-05-09 | 2009-12-28 | 2010-12-31 |
|  |  | Quasi-Identifier | Year_of_birth | | | | | 25 200 | 75 | 0.297 | 1909 | 1928 | 1936 | 1942 | 1983 |
| Categorical variable | | | | | | | |  |  |  |  |  |  |  |  |
|  | Personal Health Identifier | | Day_of_birth | | | | | 25 200 | 1 | 0.004 | - | - | - | - | - |
|  |  |  |  | Rank^e^ | | Value^f^ (%) | |  |  |  |  |  |  |  |  |
|  |  |  |  | 1 | | 1 (100%) | |  |  |  |  |  |  |  |  |
|  |  |  |  | - | | - | |  |  |  |  |  |  |  |  |
|  |  |  |  | - | | - | |  |  |  |  |  |  |  |  |
|  |  |  | County | | | | | 25 200 | 3 088 | 12.254 | - | - | - | - | - |
|  |  |  |  | Rank^e^ | | Value^f^ (%) | |  |  |  |  |  |  |  |  |
|  |  |  |  | 1 | | 5200 (17.066) | |  |  |  |  |  |  |  |  |
|  |  |  |  | 2 | | 14141 (10.719) | |  |  |  |  |  |  |  |  |
|  |  |  |  | 3 | | 3060 (8.776) | |  |  |  |  |  |  |  |  |
|  |  |  | NPI* | | | | |  |  |  | - | - | - | - | - |
|  | Quasi-Identifier | | Gender_concept_id | | | | | 25 200 | 2 | 0.008 | - | - | - | - | - |
|  |  |  |  | Rank^e^ | | | Value^f^ (%) |  |  |  |  |  |  |  |  |
|  |  |  |  | 1 | | | 8532 (55.619) |  |  |  |  |  |  |  |  |
|  |  |  |  | 2 | | | 8507 (44.381) |  |  |  |  |  |  |  |  |
|  |  |  |  | - | | | - |  |  |  |  |  |  |  |  |
|  |  |  | Race_concept_id | | | | | 25 200 | 3 | 0.012 | - | - | - | - | - |
|  |  |  |  | | Rank^e^ | Value^f^ (%) | |  |  |  |  |  |  |  |  |
|  |  |  |  |  | 1 | 8527 (83.040) | |  |  |  |  |  |  |  |  |
|  |  |  |  |  | 2 | 8516 (10.429) | |  |  |  |  |  |  |  |  |
|  |  |  |  |  | 3 | 0 (6.532) | |  |  |  |  |  |  |  |  |
|  |  |  | Ethnicity_concept_id | | | | | 25 200 | 2 | 0.008 | - | - | - | - | - |
|  |  |  |  | | Rank^e^ | Value^f^ (%) | |  |  |  |  |  |  |  |  |
|  |  |  |  |  | 1 | 38003564 (97.567) | |  |  |  |  |  |  |  |  |
|  |  |  |  |  | 2 | 38003563 (2.433) | |  |  |  |  |  |  |  |  |
|  |  |  |  |  | - | - | |  |  |  |  |  |  |  |  |
|  |  |  | Device_concept_id | | | | | 1 247 726 | 529 | 0.042 | - | - | - | - | - |
|  |  |  |  | | Rank^e^ | Value^f^ (%) | |  |  |  |  |  |  |  |  |
|  |  |  |  |  | 1 | 0 (96.181) | |  |  |  |  |  |  |  |  |
|  |  |  |  |  | 2 | 2614966 (2.160) | |  |  |  |  |  |  |  |  |
|  |  |  |  |  | 3 | 2615309 (0.269) | |  |  |  |  |  |  |  |  |
|  |  |  | Drug_concept_id | | | | | 1 300 649 | 432 | 0.033 | - | - | - | - | - |
|  |  |  |  | | Rank^e^ | Value^f^ (%) | |  |  |  |  |  |  |  |  |
|  |  |  |  |  | 1 | 0 (87.828) | |  |  |  |  |  |  |  |  |
|  |  |  |  |  | 2 | 1301125 (2.223) | |  |  |  |  |  |  |  |  |
|  |  |  |  |  | 3 | 2213440 (1.293) | |  |  |  |  |  |  |  |  |
|  |  |  | Measurement_concept_id | | | | | 1 622 884 | 851 | 0.052 | - | - | - | - | - |
|  |  |  |  | | Rank^e^ | Value^f^ (%) | |  |  |  |  |  |  |  |  |
|  |  |  |  |  | 1 | 0 (54.331) | |  |  |  |  |  |  |  |  |
|  |  |  |  |  | 2 | 2212648 (4.112) | |  |  |  |  |  |  |  |  |
|  |  |  |  |  | 3 | 2212093 (3.320) | |  |  |  |  |  |  |  |  |
|  |  |  | Observation_concept_id | | | | | 1 348 569 | 917 | 0.068 | - | - | - | - | - |
|  |  |  |  | | Rank^e^ | Value^f^ (%) | |  |  |  |  |  |  |  |  |
|  |  |  |  |  | 1 | 0 (68.783) | |  |  |  |  |  |  |  |  |
|  |  |  |  |  | 2 | 4015724 (4.332) | |  |  |  |  |  |  |  |  |
|  |  |  |  |  | 3 | 440927 (3.059) | |  |  |  |  |  |  |  |  |
|  |  |  | Procedure_concept_id | | | | | 3 105 665 | 6 300 | 0.203 | - | - | - | - | - |
|  |  |  |  | | Rank^e^ | Value^f^ (%) | |  |  |  |  |  |  |  |  |
|  |  |  |  |  | 1 | 2002291 (4.620) | |  |  |  |  |  |  |  |  |
|  |  |  |  |  | 2 | 2414397 (4.557) | |  |  |  |  |  |  |  |  |
|  |  |  |  |  | 3 | 0 (4.292) | |  |  |  |  |  |  |  |  |
|  |  |  | Condition_concept_id | | | | | 3 369 468 | 7 407 | 0.220 | - | - | - | - | - |
|  |  |  |  | | Rank^e^ | Value^f^ (%) | |  |  |  |  |  |  |  |  |
|  |  |  |  |  | 1 | 0 (4.088) | |  |  |  |  |  |  |  |  |
|  |  |  |  |  | 2 | 201826 (3.920) | |  |  |  |  |  |  |  |  |
|  |  |  |  |  | 3 | 313217 (1.957) | |  |  |  |  |  |  |  |  |
|  |  |  | Place_of_service_concept_id | | | | | 1 218 881 | 4 | 0.0003 | - | - | - | - | - |
|  |  |  |  | | Rank^e^ | Value^f^ (%) | |  |  |  |  |  |  |  |  |
|  |  |  |  |  | 1 | 8940 (84.669) | |  |  |  |  |  |  |  |  |
|  |  |  |  |  | 2 | 8756 (13.728) | |  |  |  |  |  |  |  |  |
|  |  |  |  |  | 3 | 8717 (1.430) | |  |  |  |  |  |  |  |  |
|  |  |  | State | | | | | 25 200 | 51 | 0.202 | - | - | - | - | - |
|  |  |  |  | | Rank^e^ | Value^f^ (%) | |  |  |  |  |  |  |  |  |
|  |  |  |  |  | 1 | CA (8.853) | |  |  |  |  |  |  |  |  |
|  |  |  |  |  | 2 | FL (6.536) | |  |  |  |  |  |  |  |  |
|  |  |  |  |  | 3 | TX (5.817) | |  |  |  |  |  |  |  |  |
|  |  |  | *NPI, National Provider Identifier, is a unique ID and has been excluded from categorical variables. | | | | | | | | | | | | |

^a^Number of total records refers to the total records in the PHI datasets and QI scenarios that contains certain variables.
^b^Distinct variable refers to the removal of duplicated variable values.
^c^Count denotes the number of distinct variables.
^d^Percent refers to the percentage of distinct variable number. The Percent consists of the numerator which is the number of distinct variable and the denominator which is the number of total records,
^e^Rank is the top three ranking among the categorical variables, evaluated in the order of the highest value.

^f^Value represents the top three values with the highest number of categorical variables
