# Supplementary material for: Evaluation of the Privacy Risks of Personal Health Identifiers and Quasi-Identifiers in a Distributed Research Network: Development and Validation Study
Source: JMIR Med Inform. 2021 May 31;9(5):e24940. doi: 10.2196/24940 (PMC8204238; doi:10.2196/24940)
Supplement: Multimedia Appendix 3 [file medinform_v9i5e24940_app3.docx]

Multimedia Appendix 3. Seven scenarios with personal health identifiers and quasi-identifiers in the Observational Medical Outcome Partnership common data model, based on not null values in the Synthetic Public Use File 5 Percent data set.

| **Scenario** | **Personal Health Identifier** | **Clinical variable of Quasi-Identifier** | **Personal Health Identifier** | **Demographic variable of Quasi-Identifier** |
| --- | --- | --- | --- | --- |
| Diagnosis | Condition_start_date,  Condition_end_date | Condition_concept_id | Month_of_birth,  Day_of_birth,  County,  Visit_Start_date,  Visit_end_date,  NPI | Year_of_birth,  Gender_concept_id,  Race_concept_id,  Ethnicity_concept_id,  State |
| Procedure | Procedure_date | Procedure_concept_id |  |  |
| Drug treatment | Drug_exposure_start_date,  Drug_exposure_end_date | Drug_concept_id |  |  |
| Lab Test | Measurement_date | Measurement_concept_id |  |  |
| Medical history | Observation_date | Observation_concept_id |  |  |
| Death | Death_date | Palce_of_service_concept_id |  |  |
| Device treatment | Device_exposure_start_date,  Device_exposure_end_date | Device_concept_id |  |  |
