# Supplementary material for: Evaluation of the Privacy Risks of Personal Health Identifiers and Quasi-Identifiers in a Distributed Research Network: Development and Validation Study
Source: JMIR Med Inform. 2021 May 31;9(5):e24940. doi: 10.2196/24940 (PMC8204238; doi:10.2196/24940)
Supplement: Multimedia Appendix 4 [file medinform_v9i5e24940_app4.docx]

Multimedia Appendix 4. Random sampling and 100 iterations were conducted to compare records with a minimum cell size of one between the limited and safe harbor data sets from 16 personal health identifier data sets.

| Sampling^a^ | Variable^b^ | Limited dataset | | Safe harbor dataset | | Trust differential gap^g^ (%) |
| --- | --- | --- | --- | --- | --- | --- |
|  |  | Number of minimum cell size 1^c^ | | Number of minimum cell size 1^c^ | |  |
|  |  | Mean^d^ (SD^e^) | Percent^f^ (%) | Mean^d^ (SD^e^) | Percent^f^ (%) |  |
| 1,000 |  |  |  |  |  |  |
|  | Visit_start_date | 999.26 (1.125) | 99.926 | 859.68 (16.229) | 85.968 | 13.958 |
|  | Visit_end_date | 999.78 (0.629) | 99.978 | 861.71 (15.086) | 86.171 | 13.807 |
|  | Death_date | 989.06 (2.155) | 98.906 | 890.44 (7.478) | 89.044 | 9.862 |
|  | Condition_start_date | 998.44 (1.766) | 99.844 | 857.59 (15.178) | 85.759 | 14.085 |
|  | Condition_end_date | 998.12 (2.006) | 99.812 | 858.7 (16.45) | 85.87 | 13.942 |
|  | Procedure_date | 998.24 (1.804) | 99.824 | 858.61 (16.504) | 85.861 | 13.963 |
|  | Measurement_date | 995.14 (2.971) | 99.514 | 855.11 (15.321) | 85.511 | 14.003 |
|  | Observation_date | 997.54 (2.162) | 99.754 | 854.79 (14.238) | 85.479 | 14.275 |
|  | Device_exposure_start_date | 877.05 (13.107) | 87.705 | 539.75 (16.877) | 53.975 | 33.73 |
|  | Device_exposure_end_date | 875.34 (16.05) | 87.534 | 539.68 (20.112) | 53.968 | 33.566 |
|  | Drug_exposure_start_date | 956.34 (8.669) | 95.634 | 720.7 (17.729) | 72.07 | 23.564 |
|  | Drug_exposure_end_date | 956.34 (8.669) | 95.634 | 720.7 (17.729) | 72.07 | 23.564 |
|  | Month_of_birth | 971.47 (7.612) | 97.147 | 738.4 (17.707) | 73.84 | 23.307 |
|  | Day_of_birth | 737.54 (17.774) | 73.754 | 737.54 (17.774) | 73.754 | 0 |
|  | NPI | 998.8 (1.775) | 99.88 | 673.77 (19.212) | 67.377 | 32.503 |
|  | County | 979.69 (6.59) | 97.969 | 738.46 (16.856) | 73.846 | 24.123 |
|  | average | - (-) | - | - (-) | - | 18.869 |
| 10,000 |  |  |  |  |  |  |
|  | Visit_start_date | 9 953.870 (8.749) | 99.539 | 3 683.740 (51.149) | 36.837 | 62.701 |
|  | Visit_end_date | 9 956.040 (10.311) | 99.560 | 3 675.300 (49.011) | 36.753 | 62.807 |
|  | Condition_start_date | 9 852.450 (15.789) | 98.525 | 3 624.510 (45.98) | 36.245 | 62.279 |
|  | Condition_end_date | 9 853.170 (16.953) | 98.532 | 3 623.060 (44.722) | 36.231 | 62.301 |
|  | Procedure_date | 9 827.860 (17.788) | 98.279 | 3 653.540 (54.623) | 36.535 | 61.743 |
|  | Measurement_date | 9 513.380 (29.187) | 95.134 | 3 567.800 (52.192) | 35.678 | 59.456 |
|  | Observation_date | 9 723.150 (21.763) | 97.232 | 3 597.600 (49.825) | 35.976 | 61.256 |
|  | Device_exposure_start_date | 4 423.640 (55.126) | 44.236 | 2 049.990 (31.775) | 20.500 | 23.737 |
|  | Device_exposure_end_date | 4 424.080 (56.998) | 44.241 | 2 050.650 (39.907) | 20.507 | 23.734 |
|  | Drug_exposure_start_date | 7 159.190 (56.61) | 71.592 | 2 836.400 (54.294) | 28.364 | 43.228 |
|  | Drug_exposure_end_date | 7 159.190 (56.61) | 71.592 | 2 836.400 (54.294) | 28.364 | 43.228 |
|  | Month_of_birth | 7 693.010 (49.794) | 76.930 | 2 544.810 (37.361) | 25.448 | 51.482 |
|  | Day_of_birth | 2 549.960 (37.496) | 25.500 | 2 549.960 (37.496) | 25.500 | 0 |
|  | NPI | 9 894.520 (13.122) | 98.945 | 1 509.370 (30.702) | 15.094 | 83.852 |
|  | County | 8 440.170 (40.801) | 84.402 | 2 540.740 (31.392) | 25.407 | 58.994 |
|  | average | - (-) | - | - (-) | - | 50.730 |
|  | Excluded 1 variable* |  |  |  |  |  |
| 100,000 |  |  |  |  |  |  |
|  | Visit_start_date | 95 582.700 (90.362) | 95.583 | 3 016.250 (51.935) | 3.016 | 92.566 |
|  | Visit_end_date | 95 573.240 (88.455) | 95.573 | 3 018.890 (48.862) | 3.019 | 92.554 |
|  | Condition_start_date | 86 613.360 (149.276) | 86.613 | 2 953.550 (46.083) | 2.954 | 83.660 |
|  | Condition_end_date | 86 596.050 (132.64) | 86.596 | 2 948.690 (46.422) | 2.949 | 83.647 |
|  | Procedure_date | 85 083.300 (145.839) | 85.083 | 2 975.330 (42.99) | 2.975 | 82.108 |
|  | Measurement_date | 66 439.100 (191.422) | 66.439 | 2 832.050 (45.153) | 2.832 | 63.607 |
|  | Observation_date | 77 783.200 (159.6) | 77.783 | 2 962.050 (49.587) | 2.962 | 74.821 |
|  | Drug_exposure_start_date | 40 749.860 (111.583) | 40.750 | 3 310.360 (37.012) | 3.310 | 37.440 |
|  | Drug_exposure_end_date | 40 742.140 (129.649) | 40.742 | 3 324.600 (42.905) | 3.325 | 37.418 |
|  | NPI | 92 739.740 (109.395) | 92.740 | 431.26 (18.046) | 0.431 | 92.308 |
|  | average | - (-) | - | - (-) | - | 74.013 |
|  | Excluded 6 variables* |  |  |  |  |  |
| 1,000,000 |  |  |  |  |  |  |
|  | Visit_start_date | 678 546.320 (310.626) | 67.855 | 624.95 (14.776) | 0.062 | 67.792 |
|  | Visit_end_date | 678 665.460 (339.583) | 67.867 | 543.11 (11.673) | 0.054 | 67.812 |
|  | Condition_start_date | 313 610.760 (400.354) | 31.361 | 508.91 (16.576) | 0.051 | 31.310 |
|  | Condition_end_date | 313 684.520 (421.183) | 31.368 | 473.08 (16.7) | 0.047 | 31.321 |
|  | Procedure_date | 326 911.260 (430.855) | 32.691 | 488.45 (18.103) | 0.049 | 32.642 |
|  | NPI | 735 994.550 (271.84) | 73.599 | 110.55 (6.279) | 0.011 | 73.588 |
|  | average | - (-) | - | - (-) | - | 50.744 |
|  | Excluded 10 variables* |  |  |  |  |  |

^a^Sampling isnumber of random samplings, i.e., 1000, 10 000, 100 000, and 1 million, from the limited and safe harbor datasets.

^b^Variable is variable targeted from the OMOP CDM as the PHI

^c^Number of minimum cell size 1 is the number of records with a unique record among the total records
^d^Mean is the average of the quantity with a minimum cell size 1 obtained by iterating the random sampling of each variable 100 times.
^e^SD is the standard deviation of the quantity with a minimum cell size 1 obtained by iterating random sampling of each variable 100 times. ^f^Percent is the percentage of the quantity with a minimum cell size 1. The numerator is the mean of the minimum cell size 1, which was obtained from 100 iterations, and the denominator was the number of random samples.
^g^Trust differential gap is the difference obtained by comparing two datasets to measure privacy risk.

*Some variables were excluded because the number of total records was less than the number of random samples. For 10 000 random samplings, the Death_date variable was excluded. For 100 000 random samplings, the Death_date, Day_of_birth, Month_of_birth, Device_exposure_start_date, Device_exposure_end_date, and County variables were excluded. For 1 million random samplings, the Death_date, Day_of_birth, Month_of_birth, Device_exposure_start_date, Device_exposure_end_date, County, Measurement_date, Observation_date, Drug_exposure_start_date, and Drug_exposure_end_date variables were excluded.
