# Supplementary material for: Evaluation of the Privacy Risks of Personal Health Identifiers and Quasi-Identifiers in a Distributed Research Network: Development and Validation Study
Source: JMIR Med Inform. 2021 May 31;9(5):e24940. doi: 10.2196/24940 (PMC8204238; doi:10.2196/24940)
Supplement: Multimedia Appendix 5 [file medinform_v9i5e24940_app5.docx]

Multimedia Appendix 5. Percentage of records measuring Quasi-Identifier privacy risk with gaps between the limited and safe harbor datasets with a minimum cell size of 1-5.

|  |  | Limited dataset | | | | | Safe harbor dataset | | | | | Trust differential gap,(%)^d^ | | | | |
| --- | --- | --- | --- | --- | --- | --- | --- | --- | --- | --- | --- | --- | --- | --- | --- | --- |
| Scenarios | Number of total record^a^ | Minimum cell size of 1-5 percent^b^, % (record^c^) | | | | | Minimum cell size of 1-5 percent^b^, % (record^c^) | | | | | Minimum cell size of 1-5^b^ | | | | |
|  |  | 1 | 2 | 3 | 4 | 5 | 1 | 2 | 3 | 4 | 5 | 1 | 2 | 3 | 4 | 5 |
| Diagnosis | 3,369,468 | 71.465 (2,407,996) | 11.049 (186,162) | 6.075 (68,237) | 3.729 (31,419) | 2.333 (15,726) | 41.595 (1,401,556) | 14.248 (240,043) | 8.438 (94,779) | 5.972 (50,308) | 4.412 (29,737) | 29.869 | 35.361 | 24.308 | 22.065 | 19.986 |
| Procedure | 3,105,665 | 76.123 (2,364,135) | 9.902 (153,767) | 4.49 (46,477) | 2.61 (20,261) | 1.731 (10,752) | 31.251 (970,568) | 12.472 (193,672) | 7.781 (80,559) | 5.716 (44,383) | 4.460 (27,708) | 44.871 | 42.301 | 39.009 | 35.903 | 33.173 |
| Drug treatment | 1,300,649 | 95.475 (1,241,796) | 0.895 (5,826) | 0.387 (1,679) | 0.316 (1,028) | 0.306 (796) | 5.558 (72,292) | 1.625 (10,569) | 0.748 (3,247) | 0.480 (1,563) | 0.356 (927) | 89.917 | 89.187 | 88.826 | 88.661 | 88.611 |
| Lab test | 1,622,884 | 93.012 (1,509,486) | 5.043 (40,923) | 1.214 (6,571) | 0.384 (1,559) | 0.138 (448) | 16.749 (271,819) | 7.748 (62,875) | 4.750 (25,699) | 3.199 (12,983) | 2.385 (7,744) | 76.263 | 73.558 | 70.022 | 67.206 | 64.958 |
| Medical history | 1,348,569 | 92.353 (1,245,455) | 4.286 (28,900) | 2.159 (9,706) | 0.562 (1,897) | 0.224 (606) | 12.079 (162,898) | 4.466 (30,115) | 2.753 (12,377) | 1.977 (6,666) | 1.550 (4,183) | 80.274 | 80.094 | 79.500 | 78.085 | 76.759 |
| Death | 1,218,881 | 99.997 (1,218,845) | 0.003 (18) | 0  (0) | 0  (0) | 0  (0) | 0.784 (9,557) | 0.686 (4,185) | 0.699 (2,841) | 0.703 (2,143) | 0.719 (1,755) | 99.212 | 98.529 | 0 | 0 | 0 |
| Device treatment | 1,247,726 | 97.647 (1,218,368) | 0.152 (954) | 0.055 (229) | 0.051 (161) | 0.059 (149) | 1.464 (18,271) | 0.380 (2,372) | 0.195 (814) | 0.161 (503) | 0.139 (348) | 96.182 | 95.955 | 95.814 | 95.705 | 95.625 |
| Average | - | - | - | - | - | - | - | - | - | - | - | 73.798 | 73.569 | 56.782 | 55.375 | 54.458 |

^a^Number of total records denotes each total record of the scenarios
 ^b^Minimum cell size of 1–5 percent represents the percentage of records that have a common attribute size of 1–5. This percentage is presented the records of minimum cell size of 1–5 as the numerator, and the total number of records as the denominator ^c^Record is the number of records with a common attribute size of 1–5 within the total records
^d^Trust differential gap indicates the differences obtained by comparing two datasets to measure privacy risk.
